# Why Control Activity? Evolutionary Selection Pressures Affecting the Development of Physical Activity Genetic and Biological Regulation

**DOI:** 10.1155/2013/821678

**Published:** 2013-12-24

**Authors:** J. Timothy Lightfoot

**Affiliations:** Huffines Institute of Sports Medicine and Human Performance, Health and Kinesiology Department, Texas A&M University, 356 Blocker Building, 4243 TAMU, College Station, TX 77843, USA

## Abstract

The literature strongly suggests that daily physical activity is genetically and biologically regulated. Potential identities of the responsible mechanisms are unclear, but little has been written concerning the possible evolutionary selection pressures leading to the development of genetic/biological controls of physical activity. Given the weak relationship between exercise endurance and activity levels and the differential genomic locations associated with the regulation of endurance and activity, it is probable that regulation of endurance and activity evolved separately. This hypothesis paper considers energy expenditures and duration of activity in hunter/gatherers, pretechnology farmers, and modern Western societies and considers the potential of each to selectively influence the development of activity regulation. Food availability is also considered given the known linkage of caloric restriction on physical activity as well as early data relating food oversupply to physical inactivity. Elucidating the selection pressures responsible for the genetic/biological control of activity will allow further consideration of these pressures on activity in today's society, especially the linkages between food and activity. Further, current food abundance is removing the cues for activity that were present for the first 40,000 years of human evolution, and thus future research should investigate the effects of this abundance upon the mechanisms regulating activity.

## 1. Introduction 

It has been a relatively short period of time since the first suggestions were made that physical activity may have a genetic control component [1]. Since that time, significant strides have been made in understanding the basis of both genetic and biological regulatory mechanisms of physical activity. Estimates of the amount of physical activity regulated by genetics are variable, with both human and animal studies suggesting that genetics is responsible for between 29% and 92% of activity [2, 3] with larger studies suggesting that this number is closer to 50% [4, 5]. Further, a deep and extensive set of studies have suggested that inherent biological pathways (e.g., sex steroids) have a marked control over physical activity [6–12]. Whether variability in this biological control of activity is controlled by genetics or an alternative biological mechanism is currently unclear, and thus this paper will refer to “genetic/biological” control. Thus, the purpose of this hypothesis paper is to propose a conceptual framework for considering why there might be genetic/biological regulation of physical activity and the potential selection pressures that drove the evolution of physical activity regulation. Additionally, as the scope of this paper is limited, the reader is referred to other reviews for a full discussion of the factors influencing the variability of physical activity heritability [13–17].

## 2. Exercise Endurance and Physical Activity Appear to Have Evolved Separately

It is generally accepted that *Homo sapiens* initially evolved the anatomical and physiological capability for endurance running approximately 40,000–50,000 years ago [18, 19]. However, it is unclear whether the genetic control of physical activity is a derivation from the selected traits that allowed endurance running or whether physical activity evolved as a separate trait. As we have noted elsewhere [20], it is tempting to suggest that physiological characteristics that increase endurance (e.g., mitochondrial density, fiber type) might also be key components leading to higher voluntary physical activity levels, and thus both exercise endurance and physical activity would have evolved in lockstep with each other. However, two independent lines of evidence suggest that exercise capacity and activity levels did not evolve together.

First, correlations of exercise capacity and activity levels in humans generally suggest that while the relationship is positive, there is only a low to moderate association between activity and endurance in adults (*r* = 0.01 to 0.61; [21–24]). In particular, 13 studies reviewed by Lamb and Brodie [23] demonstrated that the wide variability in the relationship between physical activity and endurance is potentially due to the use of various populations and differing recall methods for activity, as well as the use of submaximal or other indirect measures of exercise capacity. In children, the literature is not much clearer, with the largest analysis of available data (*n* = 20 studies and 53 comparisons; [25]) noting that the median relationship between physical activity and exercise capacity in children and adolescents was extremely low (*r* ^2^ value < 0.03). Results from studies using more objective measures of physical activity in children have shown higher associations in 8- to 10-year-old children (*r*-values = 0.59–0.66; [26]) and 11–18-year-old children (*r*-values = 0.031–0.393; [27]). However, in early studies relating direct measures of exercise capacity and accelerometer-based measurements of activity in adults, the association between exercise capacity and physical activity appears to be relatively weak, with males showing no correlation (*P* = 0.41, *r* ^2^ = 0.017, *n* = 42, and age = 25.2 ± 5.7 years) and females showing a significant but weak correlation (*P* = 0.003, *r* ^2^ = 0.11, *n* = 76, and age = 23.6 ± 5.2 years; [28]). Thus, while there appears to be methodological constraints on the earlier data, recent data still shows a moderate association at best between exercise capacity and activity levels with these associations dropping precipitously when used to fit prediction equations (i.e., *r* ^2^ values). Animal models, which allow the objective measurement of activity and exercise capacity without many of the environmental influences and confounds present in human models, have supported the low/moderate correlation (*r* = 0.15–0.44) between exercise capacity and daily activity levels [29–32]. Thus, even though there are methodological concerns with the data, the predominant view from the literature is that while there is a positive relationship between activity level and exercise capacity, that relationship is weak and certainly cannot be used to predict either activity level or functional capacity.

A second line of developing evidence that suggests that physical activity and exercise capacity did not evolve together involves the actual genetic mechanisms that underlie inherent exercise capacity and inherent physical activity level. While delineation of actual genetic mechanisms for either phenotype is still lacking for both animals and humans, genomic loci (i.e., QTL) associated with various indices of exercise capacity and physical activity appear to be separate and distinct [20, 33–37]. These distinct QTL suggest that genetic regulation of these traits arises through differing pathways. Thus, while there may be pathways common to both phenotypes, the low/moderate association between activity and exercise capacity in humans and animals, as well as the differing genomic loci associated with each trait, suggests that the underlying physiological regulation of inherent exercise capacity and inherent activity level of mammals evolved separately.

## 3. Potential Selection Pressures for the Evolution of Physical Activity Regulation

**Figure 1:**
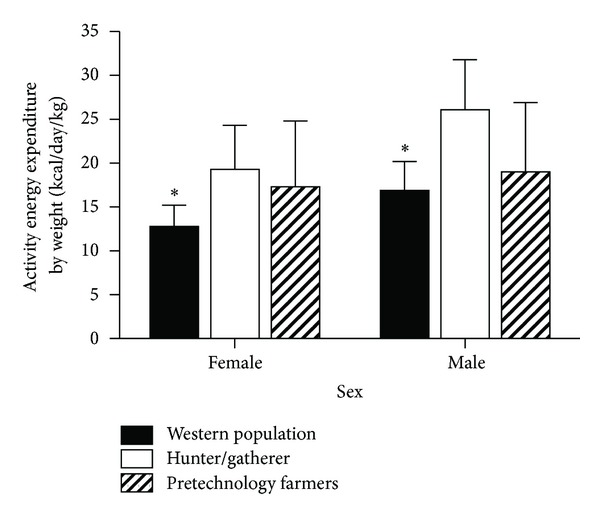
Activity energy expenditure by weight. The amount of energy expended on nonbasal activity and standardized by weight of population. Hunter/gatherer population estimates using Ju/'hoansi [39], Ache [40], and Hadza [42]. Pretechnology farmer values from populations in Figure 2 [39–41, 45, 46, 50, 54, 56, 60]. Western population data from [42]. *Significantly lower (*P* < 0.05) than Hunter/gatherer and Pretechnology farmers. There were no statistical differences between Hunter/gatherers and Pretechnology farmers. Values for Western AEE/wt used in statistical analysis derived from artificial dataset derived from means, standard deviations, and subject numbers as reported in [42].

The evolution of a physiological system is necessarily linked to genetic selection pressure [38], and the current literature is silent as to what selection pressure would have driven the evolution of systems to regulate physical activity. While hunter/gatherers were well known for having irregular, but sometimes extensive, hunting/foraging ranges [39, 40], their overall activity patterns were not uniform (e.g., [41]). Recent data have suggested that total daily energy expenditure demands (not corrected for body weight) of hunter/gatherers were not different than modern, western-based lifestyles [42, 43]. Further, comparison of energy expenditure by weight between pretechnology farmers and hunter-gatherer populations does not show significant differences in daily energy expenditures (Tables 1 and 2 and Figure 1). Whereas current Western populations show decreased energy expenditures when corrected by weight, it can be argued that the higher energy expenditure required by either hunting/gathering and/or pretechnology farming could have been a selection pressure driving the development of activity regulation. However, some investigators dispute that energy expenditure requirements have decreased [42, 43] which casts questions on the potential role that energy expenditure played in evolving activity regulation.

**Figure 2:**
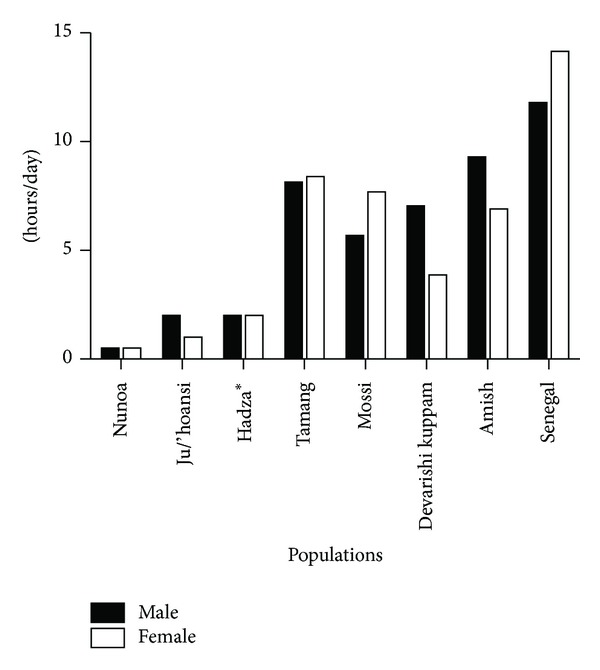
Total time spent in vigorous, moderate, or walking activity on a daily basis in hunter/gatherer (Nunoa, Ju/'hoansi, Hadza) or nontechnological agriculture-based populations (Tamang, Mossi, Devarishi Kuppam, Amish, and Senegali). Data from [39–41, 45, 46, 50, 54, 56, 60]. *Hadza activity time based on estimates from [41] which provide the only known total daily activity time estimates for this population.

While it is unclear whether energy expenditure would have been a selection pressure in the evolution of physical activity control mechanisms, comparisons of required daily activity (i.e., duration of activity) in nontechnology dependent agricultural societies (Table 2) show that the activity levels exhibited by both males and females in these populations were at least 3-fold higher than activity levels shown in hunter/gatherer populations (Figure 2). For example, Panter-Brick [44, 45] characterized a Nepali agropastoralist community (the Tamang) living at 1,350 to 3,800 m that exhibited food self-sufficiency through manual farming and livestock rearing with little to no technology use. Using both direct observations and indirect respirometry, Panter-Brick observed that the men worked an average of 8.15 ± 0.9 hours/day, while the women worked 8.4 ± 0.8 hrs/day. More recently, Bassett and colleagues [46] measured physical activity levels in a North American labor-intensive, non-technological Amish farming community. In this population, Bassett and colleagues observed that the men averaged vigorous, moderate, or walking activity for 9.3 hrs/day and women averaged 6.9 hrs/day and only sat 3.3 hr/day (13% of the day) and 2.8 hr/day (12% of the day), respectively (Figure 2). This extensive physical activity pattern was reflected in their total steps per day where the Amish men averaged 18,425 steps/day and the women averaged 14,196 steps/day. Given the extensive data from both Panter-Brick and Bassett's groups, as well as from other nontechnological farming populations (Table 2 and Figure 2), there is little doubt that non-technological subsistence farming required extensive, long-duration, and low-intensity physical activity on a daily basis.

The sustained agricultural activity requirement may not have required higher total daily energy expenditures than hunting/gathering (Figure 1), but the extensive time requirements that were 3–5-fold higher than hunting/gathering (Figure 2) would have required the physiological capability to complete lower intensities of exercise for much longer time frames than in hunting/gathering populations. The differing time requirements across which the energy was expended would have stressed different substrate systems—especially in farmers—favoring those individuals that could store and metabolize fats for longer duration activity. Thus, the ability to be physically active for long periods of the day and the requisite requirement to produce calories from fat stores could have been a significant genetic selection pressure in the development of biological/genetic control of physical activity. Further, in those early populations that adopted agriculture, individuals that were predisposed to higher levels of motivation and physical capability for daily activity would have been more successful and would have had a greater chance of reproductive success [61]. In essence, a farmer could not have been lazy and insure that his genes would be passed on to future generations because his family would not survive.

Whether the genetic selection pressure linked to the development of biological control of physical activity was energy expenditure or duration of activity, ultimately, both factors link back to the availability of food. While estimates of average hunter/gatherer foraging ranges can appear extensive (e.g., Table 1), hunter/gatherers did not range far and had reduced energy expenditure when food was close at hand. When food became difficult to get or the hunting/foraging ranges became lengthy, hunter/gatherers simply moved to more fertile sites where food was more abundant [39]. For farmers, because they were bound to a specific location, without physical activity, there was no food. In fact, food availability appears to have a direct causative effect on physical activity that is exhibited in both animal and human models, especially in the area of caloric restriction. Numerous studies report that short-term caloric restriction decreases rodent activity, but long-term caloric restriction actually increases physical activity (e.g., [62]). This same phenomenon appears in nonhuman primates (e.g., rhesus monkeys; [63]) with a concomitant increase in metabolic efficiency of movement. Further, it has been suggested that this caloric restriction-related hyperactivity also occurs in humans. Casper [64] hypothesized that, in the majority of anorexia nervosa (AN) patients that present hyperactivity (suggested to range from 38% to 70% of AN; [65, 66]), the increased activity is a result of the hypocaloric nature of AN, which differs from the lethargy seen in semistarvation states. Casper suggested several potential physiological pathways that govern this human caloric restriction and related hyperactivity. For example, Casper [64] uniquely suggests that mutations in the “foraging” gene first found in drosophila (*dgcalpha1*; [67]) can increase foraging locomotion in fruit flies [67] and honey bees [68] and may be involved in the regulation of the increased activity in AN patients. Further, the gene homologous to *dgcalpha1* in rodents and humans is “guanylate cyclase 1, soluble, alpha 2” (*GUCY1A2*), which is one of the genes that encodes soluble guanylyl cyclase (sGC), the most sensitive receptor for nitric oxide [69]. Further, the mouse homolog of *GUCY1A2* (i.e. *Gucy1A2*) is located on Chrm. 9 downstream of a known physical activity-related QTL [20]. The involvement of *GUCY1A2*, or any other genetically-based mechanism regulating activity, would support Epling and Pierce's early speculation [70] that AN patients represent a natural selection of individuals who become active during food shortages, leading to an increased chance of food finding even at the risk of negative caloric balance. Garland and Kelly [38] also suggested that individuals with higher foraging behavior could be an example of a directed natural selection. Thus, in individuals with foraging behavior more suited to the available food supply, the alleles responsible for this higher locomotor activity may be favored more highly in reproduction [38].

Conversely, to our knowledge, there have been no direct studies designed to determine if excess caloric intake directly decreases activity in human and/or animal models. Indirectly, several studies suggest that, with overfeeding, physical activity levels decrease. For example, in an elegant study, Levine and colleagues [71] showed that overfeeding both lean and obese human subjects 1,000 kcal/day above their weight maintenance needs resulted in significant decreases in free-living walking in both groups. Schmidt et al. [72] directly measured spontaneous physical activity levels (i.e., NEAT) in obesity-prone and obesity-resistant individuals and observed decreases in spontaneous physical activity in the obesity-prone individuals three days after overfeeding (but not in obesity-resistant individuals). Anecdotally, it has been observed [73] that male baboons are markedly less active (e.g., reduced climbing, laying close to sugar-source) when their caloric intake was significantly increased through the availability of a sweetened beverage containing water, high fructose corn syrup, and artificial fruit flavoring [74]. Supporting these observations are indirect results strongly suggesting, in both adults and children, that decreased physical activity was driven by an increased adiposity as opposed to adiposity being an effect of decreased activity [75, 76]. Neither Ekelund and colleagues [75] or Metcalf et al. [76] proposed potential causative biological mechanistic ties between overfeeding and inactivity, instead preferring to speculate on potential biomechanical and physical discomfort of increased weight prohibiting activity. However, other meta-analyses and animal studies have shown no relationship between body mass and activity levels (e.g., [77–79]) suggesting that it is not body weight per se decreasing activity, but rather the increase in caloric intake. Supporting this indirect evidence of a tie between overfeeding and a decrease in physical activity is a potential mechanistic pathway. It has long been known that removal of sex hormones and subsequent reduction in testosterone or estrogen levels results in large decreases in activity (e.g., [7, 80]) that can be rescued with administration of testosterone and/or estrogen which is mediated primarily through androgenic receptor pathways [6]. Recently, Bouchard et al. [81] showed conclusively that overfeeding in humans significantly decreases androgenic production—especially in males. Therefore, hypothetically, this reduction in androgenic production from overfeeding could result in a reduction in physical activity through established pathways. Thus, while it is not currently known whether increased caloric availability decreases the drive for activity, there are tentative evidence and potential hypothetical mechanisms that strongly support further research into this question.

There are some significant limitations to the preceding discussion that should be considered in interpretation of these data. In particular, the quantification of daily activity levels in both human and animals continues to undergo refinement, and the limitations of older methods should be appreciated [82]. Thus, the use of older studies that used less than optimal methods of activity measurement, such as survey or observational methods, may need to be reconsidered. For example, much of the extant hunter/gatherer activity data is based on observational or estimated activity levels and can be open to question. An example of this limitation is the recent publication of direct measures of energy expenditures collected in a Hadza population by Pontzer et al. [42] which contradict earlier observations in the Hadza which noted marked swings and inconsistencies in Hadza activity patterns [41]. Further complicating the issue of valid activity measurement is the rapid diminishing of the opportunity to collect data on peoples that represent hunter/gatherer lifestyles. Lee, who is considered the leading expert on the Ju/'hoansi, has observed the creeping influence of Western lifestyle and the diminishment of hunting/gathering in the Ju/'hoansi is due to wide access to motorized transport, other food sources, and reduction in available foraging range [39]. Thus, if modern data were collected on the Ju/'hoansi, whether this data truly represented a Paleolithic hunter/gatherer lifestyle would be a fair question—as it is with the Hadza data of Pontzer and colleagues [42]. Therefore, it is important to use the best data available in populations that best represent the target populations and we have strived to do so in this paper.

**Figure 3:**
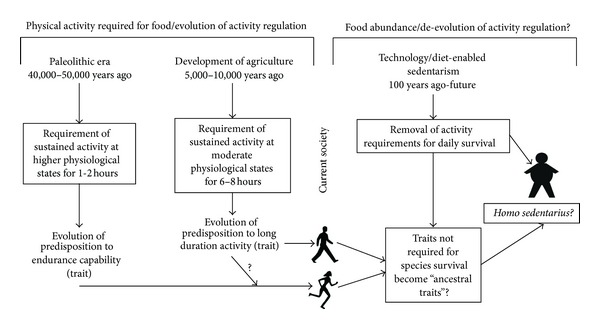
Potential selection pressures on activity regulation in humans and possible future effects of food abundance.

As scientists work to understand the identities of the genetic and biological mechanisms that control physical activity, it is important to also work to develop an understanding of the evolutionary selection pressures that have led to these activity regulation mechanisms. At this point, it is unclear what the specific genetic selection pressures were that caused the development of genetic/biological regulation of activity, but there are suggestions that physical activity evolved separately from endurance capability (Figure 3). Further, while energy expenditure may be an attractive candidate for genetic selection pressure, data suggests that total daily energy expenditure has not significantly changed, but rather, the duration of daily activity required to procure food radically changed with the adoption of agriculture approximately 10,000 years ago. Additionally, the suggestions of an inverse link between caloric intake and physical activity would add a strong biological cause/effect relationship that would both help explain evolution of genetic/biological regulation of activity and could further explain the precipitous declines in physical activity currently seen in most nations [83]. Again, the reader is cautioned that at this point in the maturity of the physical activity regulation literature, the above facts and hypotheses appear to provide the most probable—yet still hypothetical—explanation of the selection pressures influencing the evolution of physical activity regulation. Further studies directly addressing these hypotheses, especially those using animal models and experimental evolution models [38], may provide the best pathway toward conclusively establishing the evolutionary selection pressures on physical activity regulation.

## 4. Applications and Future Directions

While we have looked backward to discuss potential past causes/pressures that drove the evolution of physical activity in humans (Figure 3), it is imperative that we also look forward to consider potential areas of needed research, especially given the large health and economic consequences of the current downward trend of physical activity worldwide [83–86]. With the general acceptance of a continuing evolutionary change pattern in *Homo sapiens* amongst evolutionary biologists [87], it is interesting to speculate as to the effect of our current technology- and diet-enabled sedentarism on the genetic regulation of physical activity. As Zimmer noted in 2009 [88], predicting the outcome of evolution is difficult, especially human evolution where there are myriad factors influencing the selection of different traits. But as scientists, we should consider whether our current proclivity toward sedentarism—for example, Troiano and colleagues objectively observed less than 3.5% of adults in the United States were moderately active more than 30 mins per day [89]—will drive our evolution toward physiological mechanisms that allow us to remain inactive, yet healthy.

Theoretically, environment drives selection toward traits that increase reproductive fitness. For the first time in the history of *Homo sapiens*, we live in an era where our ability to be active or have high exercise capacity does not impact our ability to obtain food. Our current technology- and diet-enabled environment in most cases has removed the need to stay fit and be active on a daily basis. Most of us neither have to hunt and gather or grow our own food. However, since the majority of hypokinetic chronic diseases do not significantly impact health until long after the reproductive cycle of most humans has begun, as long as one can find a reproductive partner, the embracing of a technology- and diet-enabled sedentarism would not affect societal reproduction as a whole. Further, if it is assumed that the majority of individuals in a society embrace technology- and diet-enabled sedentarism, those individuals that are fit and active will become a smaller minority of the population and while potentially drawn to each other and finding health benefits in such a pairing, will find no reproductive advantage by daily exercise or activity. While the underlying genetic code that predisposes to a higher daily drive to be active will be transmitted to offspring, the environmental drive requiring the expression of that drive will be removed. Thus, in the long term, if our current technological- and diet-enabled sedentarism continues, while the mechanisms that predispose and regulate physical activity will be transmitted to our offspring, these mechanisms may fall into the category of ancestral genes that are no longer required for species survival as a whole [90]. Further, it will be interesting to observe whether genetic variants eventually evolve that enable *Homo sapiens* to physiologically deal with sedentarism—such as altered metabolic mechanisms to handle the increased fat and sugar loads characteristic of a modern diet. Whether and how *Homo sapiens* adapt and evolve for this new environment—perhaps into *Homo Sedentarius* (Figure 3)—will be an interesting topic of study and observation for years to come.

## 5. Summary and Conclusions 

Evidence suggests that daily physical activity is significantly influenced by genetic mechanisms. However, these mechanisms and the actual site of physiological regulation of physical activity at this point are somewhat unclear. This paper's goal was to provide—given the current literature—a conceptual framework that can be used to guide future investigations targeting the delineation of the genetic regulation of physical activity. First, it is unclear as to what environmental selective pressure resulted in the evolution of genetic mechanisms to control physical activity. While it is tempting to speculate that the need for ancient hunters/gatherers to run/walk long distances may have been a selective pressure, daily activity above what was needed to provide food would have put strains on energy balance within the individual and impacted the collective tribe's food supply. The acceptance of widespread agriculture demanded longer periods of activity (generally at lower intensities) and thus suggests that the longer required periods of activity inherent in farming might have provided a selection pressure. Indeed, it is often noted that lazy farmers were dead farmers. Given the known tie between food availability and activity, especially in animals, it is possible that food availability was the underlying selection pressure for the evolution of activity-regulating mechanisms. Indeed, both hunter/gatherer populations and farming populations show a negative relationship between food availability and activity. If food was scarce, activity increased and if food was available, activity decreased. Thus, food availability becomes a factor in the reason to be active. Whether food availability was the actual selection pressure for evolving regulation of physical activity is unknown but could be potentially studied given the multiple available methods of experimentally invoking evolution (e.g., [38]). The value of continued research and thought regarding the selection pressures responsible for activity regulation is to consider how modern lifestyle and food availability may impact those regulatory mechanisms. With plentiful food for the majority of the Earth's population, the requirement for physical activity to provide sustenance is markedly reduced, and thus the requirement to be physically active does not impact the survival of the species. Therefore, in the future, it will be interesting to observe whether the removal of these potential selection pressures will affect not only physical activity levels, but also the regulation of physical activity in *Homo sapiens.*

## Figures and Tables

**Table 1:** Physical activity energy expenditures of various hunter/gatherer populations.

Populations	Sex	TEE	AEE	AEE/wt	Foraging range/day (km)	Weight (kg)
Ju/'hoansi (Africa)^a,b^	M	2178	903	19.52	14.9^a^	46.0
Ju/'hoansi (Africa)^a,b^	F	1770	600	14.52	9.10^a^	41.0
Ache (Paraguay)^b^	M	3327	1778	29.75	19.2	59.6
Ache (Paraguay)^b^	F	2626	1232	24.51	9.20	51.8
Hadza (Tanzania)^c^	M	2649	1476.9	29.0	11.4	50.9
Hadza (Tanzania)^c^	F	1877	822.5	18.9	5.8	43.4

Average hunter/gatherer (±SD)	M	2718 (578)	1386.0 (444.5)	26.1 (5.7)*	15.3 (5.4)	52.2 (6.9)*
Average hunter/gatherer (±SD)	F	2091 (466)	884.8 (320.6)	19.3 (5.0)*	7.5 (2.4)	45.4 (5.7)*
Average Western population^c^	M	3053 (464)	1366.3 (268.3)	16.9 (3.3)	4.2 (2.7)^d^	81 (11.1)
Average Western population^c^	F	2347 (360)	950.0 (177.1)	12.8 (2.4)	3.2 (2.2)^d^	74.4 (12.8)

TEE: total energy expenditure (kcal/day); RMR: resting metabolic rate (kcal/day); AEE: activity energy expenditure = TEE-RMR; AEE/wt: activity energy expenditure divided by weight (kcal/kg/d); data from ^a^[39]; ^b^[40]; ^c^[42]; ^d^values calculated using average daily step counts for men and women [47] and average step lengths for men [48] and women [49]. *Significantly different *P* < 0.05 between hunter/gatherer and average western population. Values for average western population TEE, AEE, and AEE/wt used in statistical analysis derived from artificial dataset derived from means, standard deviations, and subject numbers as reported in [42].

**Table 2:** Physical activity energy expenditures of various agricultural populations.

Populations	Sex	TEE	AEE	AEE/wt	Weight (kg)
Tamang^a^ (Nepal)	M	3164	1674.3	31.3	53.5
Tamang^a^ (Nepal)	F	2382	1141.2	24.5	46.6
Devarishi Kuppam^b,c,j^ (Tamil Nadu, India)	M	2860	1580.3	31.5	50.2
Devarishi Kuppam^b,c,j^ (Tamil Nadu, India)	F	1984	902.3	20.6	43.8
Gambian^b,k^ (Gambia)	M	2292	716.4	12.3	58.47
Gambian^b,d,e^(Gambia)	F	2480	1178.45	23.73	49.7
Mossi^b,f^ (Upper Volta)	M	2913	920.51	15.74	58.5
Mossi^b,g^ (Upper Volta)	F	2603	822.55	16.25	50.6
Senegal^b,h^ (Senegal)	M	2538	901.25	13.78	65.4
Senegal^b,h ^(Senegal)	F	2573	1219.75	21.10	57.8
Amish^i^ (Canada)	M	3100	1292.3	17.65	73.2
Amish^i^ (Canada)	F	1850	304.04	4.85	62.6
Aymara^l^ (Bolivia)	M	2329	1299.4	23.7	54.8
Aymara^l^ (Bolivia)	F	2654	1184.2	24.4	48.6

Average farming populations (±SD)	M	2742 (357)	1197.9 (362.7)	20.8 (8.1)^†^	59.2 (7.8)^†^
Average farming populations (±SD)	F	2361 (318)	964.6 (329.3)	19.3 (7.0)^†^	51.4 (6.6)^†^

TEE: total energy expenditure (kcal/day; average between dry and wet season where available); RMR: resting metabolic rate (kcal/day); AEE: activity energy expenditure =TEE-RMR; AEE/wt: activity energy expenditure divided by weight in kcal/kg/d; data from ^a^[44, 45]; ^b^reviewed by [45]; data from ^c^[50]; ^d^[51]; ^e^[52]; ^f^[53]; ^g^[54]; ^h^[55]; ^i^[46]: BMRs estimated using formula (3.5 mL/kg/min O_2_) ∗ 4.9; ^j^[56]; ^k^[57] values derived from Ph.D. thesis [58]; ^l^[59]. ^†^Significantly different *P* < 0.05 between farming and Western populations. Values for Western TEE, AEE, and AEE/wt used in statistical analysis derived from artificial dataset derived from means, standard deviations, and subject numbers as reported in [42]. No significant differences between hunter/gatherer and Farming populations.
